# [^18^F]-sodium fluoride autoradiography imaging of nephrocalcinosis in donor kidneys and explanted kidney allografts

**DOI:** 10.1038/s41598-021-81144-4

**Published:** 2021-01-19

**Authors:** Stan Benjamens, Ines F. Antunes, Jan-Luuk Hillebrands, Melanie Reijrink, Marian L. C. Bulthuis, Stefan P. Berger, Cyril Moers, Martin H. de Borst, Riemer H. J. A. Slart, Robert A. Pol

**Affiliations:** 1 Department of Surgery, Division of Transplant Surgery, University Medical Center Groningen, University of Groningen, P.O. Box 30 001, 9700 RB Groningen, The Netherlands; 2 Department of Nuclear Medicine and Molecular Imaging, Medical Imaging Center, University Medical Center Groningen, University of Groningen, Groningen, The Netherlands; 3 Department of Pathology and Medical Biology, Pathology Section, University Medical Center Groningen, University of Groningen, Groningen, The Netherlands; 4 Department of Vascular Medicine, University Medical Center Groningen, University of Groningen, Groningen, The Netherlands; 5 Department of Internal Medicine, Division of Nephrology, University Medical Center Groningen, University of Groningen, Groningen, The Netherlands; 6 Department of Biomedical Photonic Imaging, Faculty of Science and Technology, University of Twente, Enschede, The Netherlands

**Keywords:** Diagnostic markers, Kidney

## Abstract

Nephrocalcinosis is present in up to 43% of kidney allograft biopsies at one-year after transplantation and is associated with inferior graft function and poor graft survival. We studied [^18^F]-sodium fluoride ([^18^F]-NaF) imaging of microcalcifications in donor kidneys (n = 7) and explanted kidney allografts (n = 13). Three µm paraffin-embedded serial sections were used for histological evaluation of calcification (Alizarin Red; Von Kossa staining) and ex-vivo [^18^F]-NaF autoradiography. The images were fused to evaluate if microcalcification areas corresponded with [^18^F]-NaF uptake areas. Based on histological analyses, tubulointerstitial and glomerular microcalcifications were present in 19/20 and 7/20 samples, respectively. Using autoradiography, [^18^F]-NaF uptake was found in 19/20 samples, with significantly more tracer activity in kidney allograft compared to deceased donor kidney samples (*p* = 0.019). Alizarin Red staining of active microcalcifications demonstrated good correlation (Spearman’s rho of 0.81, *p* < 0.001) and Von Kossa staining of consolidated calcifications demonstrated significant but weak correlation (0.62, *p* = 0.003) with [^18^F]-NaF activity. This correlation between ex-vivo [^18^F]-NaF uptake and histology-proven microcalcifications, is the first step towards an imaging method to identify microcalcifications in active nephrocalcinosis. This may lead to better understanding of the etiology of microcalcifications and its impact on kidney transplant function.

## Introduction

Nephrocalcinosis, defined as parenchymal and tubular deposition of calcium-oxalate (CaOx) or calcium-phosphate (CaPhos), is an important factor in the decline of graft function after kidney transplantation^1–3^. Deposition of CaOx and CaPhos are attributed to high levels of serum oxalate, serum calcium and serum phosphate in patients with chronic kidney disease and/or hyperparathyroidism^4,5^. The incidence of nephrocalcinosis, based on kidney allograft biopsies, is 6% at 6 months, 25–43% at one-year and up to 79% at 10-years after kidney transplantation^5–7^. Studies focusing on kidney allograft biopsies within one-year after transplantation demonstrated an association between the early presence of calcium depositions and both inferior graft function and poor graft survival^4,8,9^. However, whether these microcalcifications play an active role in allograft dysfunction or are merely indicators of deregulated mineral metabolism remains unclear.

To date, kidney allograft biopsies are the only diagnostic method to evaluate post-transplant microcalcifications. A non-invasive and reliable diagnostic approach would allow for repeated measurements, facilitating tracking of calcifications over time and intervention studies with potential improvement of outcomes for graft function and graft survival after kidney transplantation^10^.

Positron emission tomography/computed tomography (PET/CT) imaging with [^18^F]-sodium fluoride ([^18^F]-NaF) is used in clinical practice and experimental studies as skeletal and vascular imaging modality^11–15^. While reliable visualization of vascular microcalcifications is hampered with CT imaging, results of ex-vivo [^18^F]-NaF microPET imaging correlated well with histology-proven microcalcifications in human and animal vascular samples^16,17^. Moreover, the results of several prospective clinical studies showed that early vascular [^18^F]-NaF activity relates well to both macrocalcifications, detected by CT imaging, and more interestingly, the increase in size of these calcifications^18–20^.

Here, we present the results of a proof of concept autoradiography study for ex-vivo use of [^18^F]-NaF imaging of nephrocalcinosis in donor kidneys and transplanted kidney allografts as a diagnostic approach for the visualization of microcalcifications.

## Results

### Sample cohort

Thirteen kidney samples were obtained after transplantectomy, at a median of 33 [IQR 9–66] months after transplantation, from transplant recipients with a median age of 51 [IQR 30–64] years. Transplantectomy was performed for allograft rejection (Banff IIA and IIB) in seven patients, chronic allograft arteriopathy in two patients, allograft infection in two patients, primary nonfunction one patient, and interstitial fibrosis and tubular atrophy Grade III in one patient. Eight out of 13 (61.5%) patients had hyperparathyroidism, seven (53.8%) had hyperphosphatemia, five (38.5%) had diabetes mellitus and none of these patients had signs of nephrocalcinosis on clinical ultrasound or a history of kidney stones (Table 1). Seven deceased donor kidney samples were obtained, from donors with a median age of 65 [IQR 64–71] years. Four out of seven (57.1%) donors had a history of cardiovascular disease, none had diabetes mellitus and six out of seven (85.7%) had atherosclerosis of the kidney vasculature on visual inspection, which are potential factors for declining these donor kidneys (Table 2).

**Table 1:** Kidney transplant recipient characteristics.

	Age	Gender	Months post-transplant	Hyperpara-thyroidism	Hyper- phosphatemia	Diabetes mellitus	Pathology assessment*
I	67	Male	7	No	No	Yes	Primary non-function
II	21	Male	35	Yes	Yes	No	Vascular rejection (type IIB)
III	43	Female	13	No	No	Yes	Vascular rejection (type IIA) & infection
IV	30	Female	74	No	Yes	Yes	Interstitial fibrosis & cellular rejection (type IIA)
V	27	Female	10	Yes	No	No	Vascular rejection (type IIB) & ischemic nephropathy
VI	57	Male	6	Yes	Yes	No	FSGS & vascular rejection (type IIB) & CaOx depositions
VII	28	Male	43	Yes	Yes	No	Chronic allograft arteriopathy
VIII	78	Male	1	Yes	Yes	Yes	IFTA Grade III
IX	64	Female	146	Yes	No	No	Chronic allograft arteriopathy & IFTA Grade III
X	37	Male	31	No	Yes	No	Acute rejection (type IIB) & IFTA Grade III
XI	52	Female	215	Yes	Yes	Yes	Infection & extensive ischemic damage
XII	37	Female	55	Yes	No	No	Acute rejection (type IIB)
XIII	63	Female	22	No	No	No	Infection & extensive ischemic damage

No cases of hypercalcemia is this cohort; *classification of Banff Classification of Allograft Pathology—2018^28^; FSGS = Focal segmental glomerulosclerosis; IFTA = Interstitial fibrosis and tubular atrophy; corresponding autoradiography and histology results are presented in Supplementary S1.

**Table 2:** Deceased kidney donor characteristics.

	Age	Gender	Cause of death	Cardiovascular disease*	Atherosclerosis**
I	71	Male	Cardiac arrest	Yes	Massive
II	63	Male	Intra cerebral bleeding	Yes	Massive
III	47	Male	Cardiac arrest	No	Unknown
IV	71	Male	Cardiac arrest	No	Moderate
V	65	Male	Intra cerebral bleeding	Yes	Massive
VI	71	Female	Sub Arachnoid bleeding	Yes	Massive
VII	54	Male	Traumatic brain injury	No	Moderate

*Cardiovascular disease defined as cerebrovascular event, myocardial infarction, vascular disease requiring invasive intervention; **as noted by the procurement team; Corresponding autoradiography and histology results are presented in Supplementary S2; no donors with diabetes mellitus.

### Autoradiography and histology

**Figure 1:**
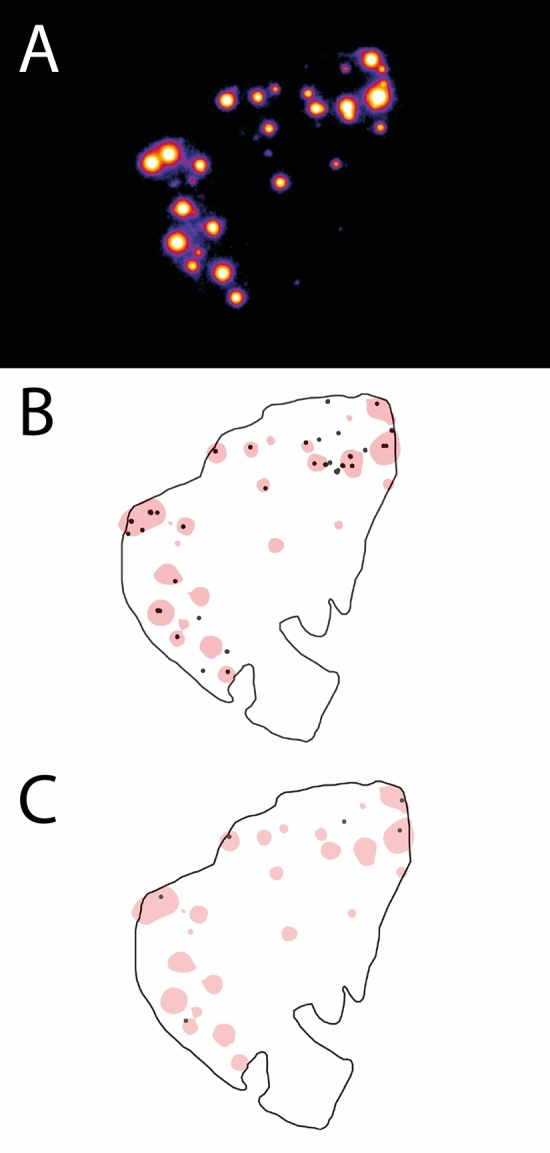
Autoradiography image of [^18^F]-sodium fluoride ([^18^F]-NaF) uptake (**A**), with the overlay image of [^18^F]-NaF uptake with Alizarin red staining of microcalcifications (**B**) and Von Kossa staining of calcifications (**C**).

**Figure 2:**
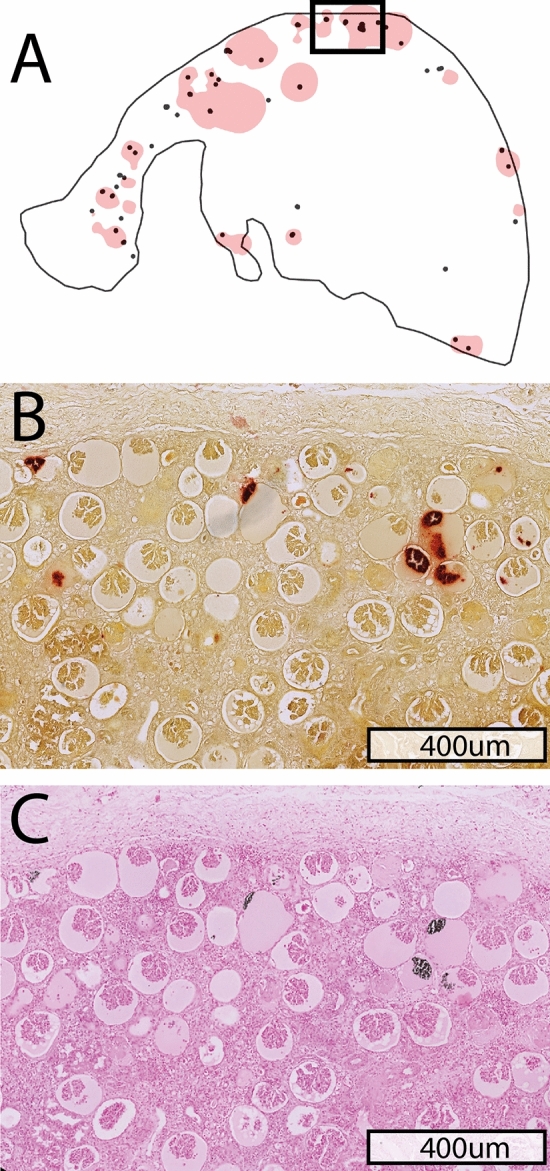
Overlay image of [^18^F]-sodium fluoride ([^18^F]-NaF) uptake and (Alizarin red) microcalcifications (**A**), with Alizarin red staining for microcalcifications (**B**) and Von Kossa staining for calcifications (**C**).

With autoradiography, [^18^F]-NaF uptake was identified in 19 out of 20 samples, with a median tracer surface area of 0.81% [IQR 0.27–5.19%]. A tracer surface area of ≤ 5% was seen in 15 (75%) and of > 5% in 5 (25%) out of 20 samples (Fig. 1 and Fig. 2A) (Supplementary S1 and Supplementary S2, showing the autoradiography and overlay images for all samples). The number of [^18^F]-NaF areas was significantly higher in kidney allograft samples compared to deceased donor kidney samples, with a median of 7.0 [IQR 4.0–15.5] and 2.0 [IQR 1.0–6.0], respectively (*p* = 0.019) (Supplementary S3).

Tubulointerstitial microcalcifications, visualized with Alizarin red staining, were present in 19 out 20 samples, with a median of 5.5 [IQR 2–10.3] per sample, and glomerular microcalcifications were seen in seven out 20 samples, with a median of 1 [IQR 1–3] per sample (Fig. 2B). A significantly higher number of microcalcifications was observed in kidney allograft samples compared to deceased donor kidney samples, with a median of 9.0 [IQR 5.0–20.5] and 4.0 [IQR 1.0–4.0], respectively (*p* = 0.046) (Supplementary S3). With Von Kossa staining, tubulointerstitial calcifications were seen in 18 out 20 samples, with a median of 2.5 [IQR 1–5] per sample, and glomerular calcifications were seen in seven out 20 samples, with a median of 1 [IQR 0–3] per sample (Fig. 2C). The number of calcifications was not significantly different between kidney allograft samples and deceased donor kidney samples, with a median of 4.0 [IQR 2.0–6.0] and 2.0 [IQR 2.0–7.0], respectively (*p* = 0.311) (Supplementary S3). The interobserver reproducibility was identified between visual and semi-automated detection of calcifications, with an ICC of 0.94 (95% CI 0.85–0.98) for Alizarin red staining and of 0.77 (95% CI 0.42–0.91) for Von Kossa staining.

**Figure 3:**
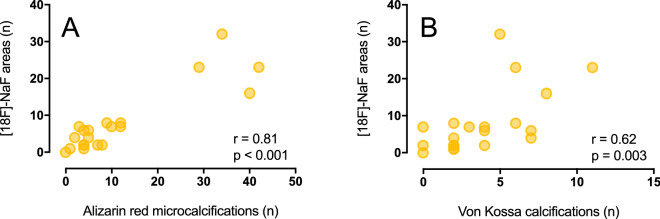
Correlation of the number of [^18^F]-sodium fluoride ([^18^F]-NaF) areas with Alizarin red stained microcalcifications (**A**) and Von Kossa stained calcifications (**B**).

**Figure 4:**
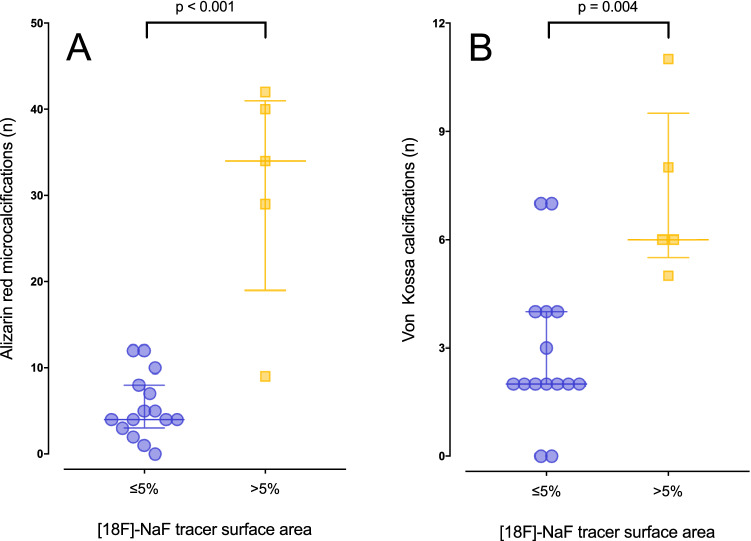
Differences between two groups, ≤ 5% and > 5% [^18^F]-sodium fluoride ([^18^F]-NaF) tracer surface area, for the number of Alizarin red stained microcalcifications (**A**) and Von Kossa stained calcifications (**B**) (median, interquartile range).

Overlay images of autoradiography and histology results showed co-localization of [^18^F]-NaF uptake with Alizarin red staining for microcalcification (Fig. 1B). In comparison, overlay images of [^18^F]-NaF uptake and Von Kossa staining of calcifications showed uptake in areas without histology-proven calcifications (Fig. 1C). The false negative [^18^F]-NaF uptake, as the percentage of histology-proven (micro)calcification areas outside the overlay, was median 0% [IQR 0–25%] for Alizarin red areas and median 100% [65–100%] for Von Kossa areas. Alizarin Red staining of microcalcifications demonstrated good correlation with [^18^F]-NaF uptake (Spearman’s rho of 0.81 (95% CI 0.57–0.92), *p* < 0.001), whereas Von Kossa staining demonstrated weak correlation with [^18^F]-NaF uptake (0.62 (95% CI 0.24–0.84), *p* = 0.003) (Fig. 3). Significant differences in the number of (micro)calcifications, by Alizarin red (median 4.0 [IQR 3.0–8.0] versus 34.0 [IQR 19.0–41.0], *p* < 0.001) and Von Kossa (median 2.0 [IQR 2.0–4.0] versus 6.0 [5.5–9.5], *p* = 0.004) staining, were observed between samples with ≤ 5% and > 5% [^18^F]-NaF uptake (Fig. 4).

### Recipient characteristics at time of transplantectomy

No differences were observed between kidney allograft samples from patients with (n = 7) or without hyperphosphatemia (n = 6), for the number of [^18^F]-NaF areas (median 5.0 [IQR 2.0–11.8] versus 7.0 [7.0–23.0], *p* = 0.170), Alizarin red (median 7.5 [IQR 5.0–14.0] versus 12.0 [3.0–34.0], *p* = 0.390), and Von Kossa (median 5.0 [IQR 3.5–6.3] versus 3.0 [2.0–5.0], *p* = 0.247). Comparing kidney allograft samples from patients with (n = 8) or without (n = 5) hyperparathyroidism did not show differences, for the number of [^18^F]-NaF areas (median 7.0 [IQR 4.0–7.5] versus 7.5 [4.0–23.0], *p* = 0.459), Alizarin red (median 9.0 [IQR 6.0–11.0] versus 10.0 [3.5–32.8], *p* = 0.769), and Von Kossa (median 4.0 [IQR 1.5–5.0] versus 4.5 [2.0–6.8], *p* = 0.505). Significant differences were not identified between kidney allograft samples from patients with (n = 5) or without (n = 8) diabetes mellitus, for the number of [^18^F]-NaF areas (median 5.5 [IQR 2.5–19.3] versus 7.0 [6.5–15.5], *p* = 0.524), Alizarin red (median 10.0 [IQR 5.5–24.8] versus 9.0 [4.0–8.5], *p* = 0.833), and Von Kossa (median 3.0 [IQR 2.0–5.8] versus 4.0 [3.5–8.5], *p* = 0.284). For the time between transplantation and transplantectomy (months) no significant correlations were observed with the number of [^18^F]-NaF areas (*p* = 0.586), Alizarin red (*p* = 0.667) and Von Kossa (*p* = 0.459). For the age at time of transplantation no significant correlations were observed with the number of [^18^F]-NaF areas (*p* = 0.902), Alizarin red (*p* = 0.798) and Von Kossa (*p* = 0.334). Similarly, no significant correlations were observed between the dialysis duration (months) after kidney allograft failure and number of [^18^F]-NaF areas (*p* = 0.481), Alizarin red (*p* = 0.565) and Von Kossa (*p* = 0.174).

## Discussion

In this proof of concept study, ex-vivo [^18^F]-NaF uptake in human kidney parenchyma samples demonstrated good correlation with histology-proven microcalcifications. Uptake of [^18^F]-NaF correlated stronger with Alizarin red staining for active microcalcifications compared to Von Kossa staining of consolidated calcifications, suggesting a role for [^18^F]-NaF imaging in the identification of early stage areas of nephrocalcinosis.

The presented samples represent a case mix of donor kidneys and explanted kidney allografts. These two sample types differed with regard to tracer binding, with more [^18^F]-NaF uptake observed in explanted kidney allograft samples. This is in line with previous publications, reporting an increase of microcalcifications after kidney transplantation^5–7^. The association of nephrocalcinosis with inferior graft function at time of biopsy and at one-year after transplantation is reported in two kidney allograft biopsy studies, with 258 and 149 kidney transplant recipients respectively^8,9^. Moreover, in a longitudinal cohort with 97 kidney transplant recipients, a significantly worse 1-year and 12-year graft survival is shown in patients with calcium-oxalate depositions (i.e. a graft survival of 98.6% versus 72.5% and 79.3% versus 49.7%, respectively). In multivariate logistic regression analysis, the presence of calcium-oxalate depositions was independently associated with graft loss, in a model including among others creatinine at time of biopsy and donor type^4^. The specific role of microcalcifications in this cascade of kidney allograft function decline remains unclear. In the pre-transplantation clinical setting, CaOx depositions are attributed to high levels of serum oxalate, a molecule not efficiently removed during dialysis^4^. Whereas CaPhos depositions are most commonly seen in patients with hyperparathyroidism and high levels of serum calcium, due to a disordered mineral metabolism pre- and post-transplantation^5^. We found no differences in histology and autoradiography results for hyperphosphatemia, hyperparathyroidism, diabetes mellitus, time between transplantation and transplantectomy and the dialysis duration after kidney allograft failure.

For the development of novel treatment strategies for active microcalcifications in nephrocalcinosis, its clinical relevance with regard to (chronic) transplant failure should be understood more comprehensively. With the identification of a target population of high-risk kidney transplant recipients, clinical studies can be designed to explore treatment strategies^21^. A possible focus could be found in the up-regulation of endogenous calcification inhibitors, with a specific emphasis on osteopontin^3^.

Given the high incidence of nephrocalcinosis and its association with kidney allograft function, better understanding of the etiology of microcalcifications is warranted. A non-invasive and reliable diagnostic approach to identify and quantify microcalcifications in early nephrocalcinosis could enable studies on impact of nephrocalcinosis on kidney transplant function. [^18^F]-NaF imaging is of interest, since [^18^F]-NaF uptake in human and animal vascular samples is increased in biologically active areas of calcification, before these areas can be visualized by micro-computed tomography^16,17,22^. In recent clinical PET/CT studies, [^18^F]-NaF uptake demonstrated to be related to the progression of vascular calcification, providing clinical evidence for the use of [^18^F]-NaF for detecting areas with biologically active calcification^18–20^. The observations in this study are in line with these previous findings in the field of vascular imaging. A good correlation between [^18^F]-NaF uptake and the histology-proven microcalcifications was demonstrated, while the correlation with consolidated calcifications was clearly weaker.

As stated, co-localization between [^18^F]-NaF uptake and Alizarin red positive microcalcifications was identified, while [^18^F]-NaF uptake areas did not co-localize with Von Kossa staining of consolidated calcifications. These calcified areas, with consolidated calcifications and no corresponding [^18^F]-NaF uptake might be considered areas of inactive calcification, where there is no ongoing process of mineralization, as earlier described by Irkle et al. in an ex-vivo study of carotid calcification^16^. Although only validated for vascular calcifications and not for kidney parenchyma calcifications, it is supported by the binding properties of [^18^F]-NaF to the outer surface of calcifications and limited penetration in solid calcifications. Therefore, [^18^F]-NaF uptake will be higher in microcalcifications, with a large surface area, compared to macrocalcifications, with a high volume but relatively small surface area^13,16^.

This proof of concept study also has some limitations. First, inherent to the study design, the results represent a single time point, lacking clinical follow-up of calcification progression. Second, this proof of concept study consists of a relatively low number of kidney samples, as transplantectomy after late transplant failure is only performed on clinical indication and the availability of declined donor kidneys for research use is limited^23^. Therefore, a comprehensive analysis of the etiology of calcium depositions should be considered beyond the scope of this project. Third, clinical translation of the results is hampered by the lack of knowledge on in-vivo [^18^F]-NaF distribution in kidneys and the effect of kidney tracer clearance, i.e. ﻿a low target-to-background activity could limit visualization of calcifications. To tackle this problem, the optimal moment of [^18^F]-NaF PET imaging should be assessed, i.e. at which [^18^F]-NaF is renally excreted and remaining activity comes from binding to calcified areas. Possibly, this can be achieved with delayed imaging exceeding three hours, as performed for assessment of coronary calcifications^24^. The reduced glomerular filtration rate found in transplant recipients could result in a prolonged excretion phase, requiring a longer period between tracer injection and PET imaging, but current guidelines do not indicate potential nephrotoxicity of the [^18^F]-NaF tracer^11,25^. A potential step between the here presented proof of concept study and in-vivo imaging can be ex-vivo [^18^F]-NaF PET imaging of normothermic perfused kidneys or in-vivo [^18^F]-NaF microPET animal studies^26,27^. Fourth, clinical application of this technique could be hampered by the costs for performing a [^18^F]-NaF PET/CT procedure and the inherent radiation exposure. However, the new digital PET camera systems with a higher sensitivity may reduce both, due to shorter scanning time and lower injected activity dose.

The strength of this proof of concept study lies in the detailed one-to-one comparison of microcalcifications identified by [^18^F]-NaF autoradiography and histology. The [^18^F]-NaF uptake in different samples could be compared without need for further adjustments, as all samples were incubated in the same [^18^F]-NaF solution. Moreover, this study provides the first data showing a visual and statistical match between [^18^F]-NaF uptake and histology-proven microcalcifications in kidney samples.

To conclude, we provide the first data for the use of [^18^F]-NaF in an autoradiography study to identify microcalcifications in active nephrocalcinosis after kidney transplantation. This ex-vivo [^18^F]-NaF proof of concept study is the first step towards the potential application of clinical [^18^F]-NaF PET/CT in kidney transplant recipients.

## Methods

### Human kidney samples

Kidney tissue was obtained after transplantectomy (n = 13) and from discarded kidneys from deceased donors (n = 7) in case of decline of the donor kidney by affiliated transplant centers. Clinical data from kidney transplant recipients and donors was retrieved from the patients’ medical records, including presence of hyperparathyroidism, hyperphosphatemia and diabetes mellitus prior to transplantectomy, and clinical pathology assessment of kidney allograft samples reported using the *Banff Classification of Renal Allograft Pathology*^28^. Research activities were performed in line with the declaration of Helsinki Ethical principles for medical research involving human subjects and the Institutional review board gave approval for this study (Medical Ethical Committee *University Medical Center Groningen* 2015/501). Informed consent was obtained from all transplant recipients and pre-donation approval was given for the use of discarded kidneys from deceased donors for research purposes. The clinical and research activities were consistent with the Principles of the Declaration of Istanbul as outlined in the 'Declaration of Istanbul on Organ Trafficking and Transplant Tourism’.

The Standard Operating Procedure (SOP) for tissue sampling was as follows: Samples were obtained at surgical theatre, immediately following kidney extraction (for transplantectomy) or at arrival of the discarded donor kidney in our transplant center. The samples used for this experiment were taken in the transverse plane of the upper pole, including cortical and medullar kidney tissue, with a size between 25 mm × 15 mm and 20 mm × 40 mm. These samples were formalin-fixed and paraffin-embedded. Using a microtome precision cutting instrument, three µm paraffin-embedded sections made. These sections were mounted on adhesion microscope glass (TOMO, Matsunami Glass, USA). Serial sections, i.e. adjacent sections that reveal sequential layers of the kidney tissue samples, were used for autoradiography and histological analysis. Prior to tracer incubation and histological staining, sections were de-paraffinized, rehydrated in demineralized water, and temporarily stored in phosphate-buffered saline (PBS).

### [^18^F]-NaF incubation and autoradiography

The ex-vivo experiment was based on the protocol used in the previous studies by Hop et al. and Reijrink et al.^22,29^. The first study is an autoradiography study on [^18^F]-NaF uptake in carotid plaques from stroke patients undergoing surgery, with CT-based indication of calcification as comparison. The second study is an autoradiography study on [^18^F]-FDG uptake in adipose tissue samples, with immunohistochemistry of serial sections as comparison. One of each three serial sections was bathed in a solution of [^18^F]-NaF, at a concentration of 618 millibecquerel (MBq) in 300 mL PBS, at room temperature, for 30 min. Washing was performed by three times rinsing with cold PBS and twice with cold water. Samples were dried in open air and a blanked phosphor screen was placed over the samples. Exposure was performed overnight, for a period of 24 h and 30 min. Determining tissue activity distribution and intensity was performed on a phosphor screen using the Amersham Typhoon Biomolecular Imager (GE healthcare, Marlborough, USA).

### Alizarin red and Von Kossa staining

Alizarin red staining was performed to visualize calcium accumulation in early stage calcification. Samples were incubated in 2% w/v Alizarin red (dissolved in demineralized water) solution, pH 4.2 for 5 minutes^30^. Washing was performed by 20 times rinsing in 1:1 acetone/xylene and 20 times rinsing in 100% xylene. Von Kossa staining was performed to visualize presence of inorganic phosphate molecules in consolidated calcifications. Briefly, samples were incubated in 1% silver nitrate (AgNO_3_) solution for 1 h, while being exposed to sunlight^30^. Alternated by three times rinsing in demineralized water, incubation was performed with sodium thiosulfate for 5 min, followed by incubation in nuclear fast red for 3 min.

### Image processing and analyses

Stained tissue sections were digitalized with the Hamamatsu Nanozoomer 2.0HT (Hamamatsu Photonics, Hamamatsu, Japan), and images were inspected using Aperio ImageScope software version 12.1.0.5029 (Leica Microsystems CMS GmbH, Wetzlar, Germany). Autoradiography and quantitative computerized image analyses were performed with ImageJ software, version 1.51 (NIH, Bethesda, Maryland, http://fiji.sc).

Background subtraction was applied for all histology images, inserting an outline at the border of the histology sample. Semi-automated detection of calcifications in the Alizarin red and Von Kossa staining was performed with the ImageJ—BioVoxxel Toolbox^31^. Thresholds were applied for all samples and detected areas of calcification were dilated with 6 iterations, to optimize visualization. For the autoradiography images, rotation and re-sizing was performed based on the original histology samples. Thresholds were applied for all samples, resulting in areas of tracer uptake, following the approach described by Irkle et al.: images were thresholded using the Otsu method, followed by Gaussian blurring and thresholding with the Li method^16^. Overlay images were acquired by matching of the outlines from the adjacent histology images, with subsequent matching of the histology and autoradiography results (Fig. 1). Pixel-based quantification of calcification and tracer area (as percentage of the sample area) was performed with the ImageJ—Analyze Particles tool.

### Statistical analyses

Statistics were performed with the Statistical Package for the Social Sciences version 23 (IBM Corporation, Armonk N.Y., USA), graphs were made with GraphPad Prism 7.02 for Windows (GraphPad Software, San Diego, USA) and figures were produced using Adobe Illustrator CS6 (Adobe Systems, San Jose, USA). Results were presented as median and interquartile range (IQR) for skewed data, and as frequency and percentage when data were categorical. We compared groups using Mann–Whitney U tests (two-tailed *p* value < 0.05 = significant) and correlation analyses were performed using the Spearman rank test (two-tailed p-value and Spearman’s rho). True and false positive rates were based on the pixel-based quantification of tracer and histology findings. To assess the interobserver reproducibility for semi-automated detection of calcifications, the intraclass correlation coefficient (ICC) with 95% confidence intervals (CI) were calculated.

## Supplementary Information

Supplementary Figures.

## Data Availability

Deceased donor kidney and kidney transplant recipient autoradiography images of [^18^F]-sodium fluoride ([^18^F]-NaF) uptake, with the overlay images of [^18^F]-NaF uptake with Alizarin red staining of microcalcifications and Von Kossa staining of calcifications are online available as Supplementary Information. All additional data will be made available on request, in line with the IRB regulations of the University Medical Center Groningen.
