# Supplementary material for: [18F]-sodium fluoride autoradiography imaging of nephrocalcinosis in donor kidneys and explanted kidney allografts
Source: Sci Rep. 2021 Jan 19;11:1841. doi: 10.1038/s41598-021-81144-4 (PMC7815841; doi:10.1038/s41598-021-81144-4)

Supplementary Information

Title

[^{18}F]-sodium fluoride autoradiography imaging of nephrocalcinosis

in donor kidneys and explanted kidney allografts

Authors

Stan Benjamens, Bsc, Ines F Antunes, PhD, Jan-Luuk Hillebrands, PhD,

Melanie Reijrink, Bsc, Marian L.C. Bulthuis, Stefan P. Berger, MD, PhD, Cyril Moers, MD, PhD,

Martin H. de Borst, MD, PhD, Riemer H.J.A Slart, MD, PhD, Robert A. Pol, MD, PhD

Supplementary S1
Kidney transplant recipient samples

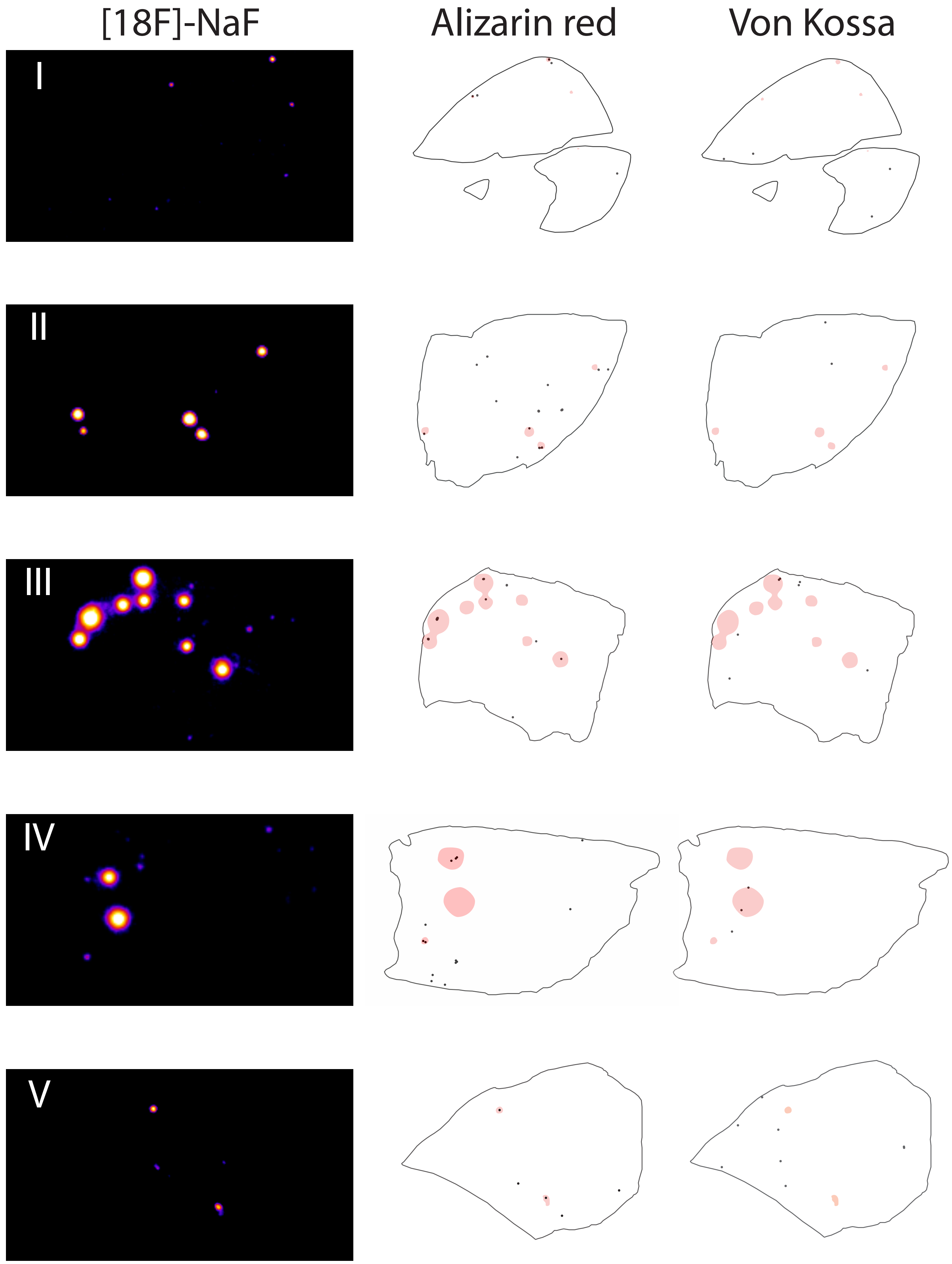

Supplementary S1

Kidney transplant recipient samples

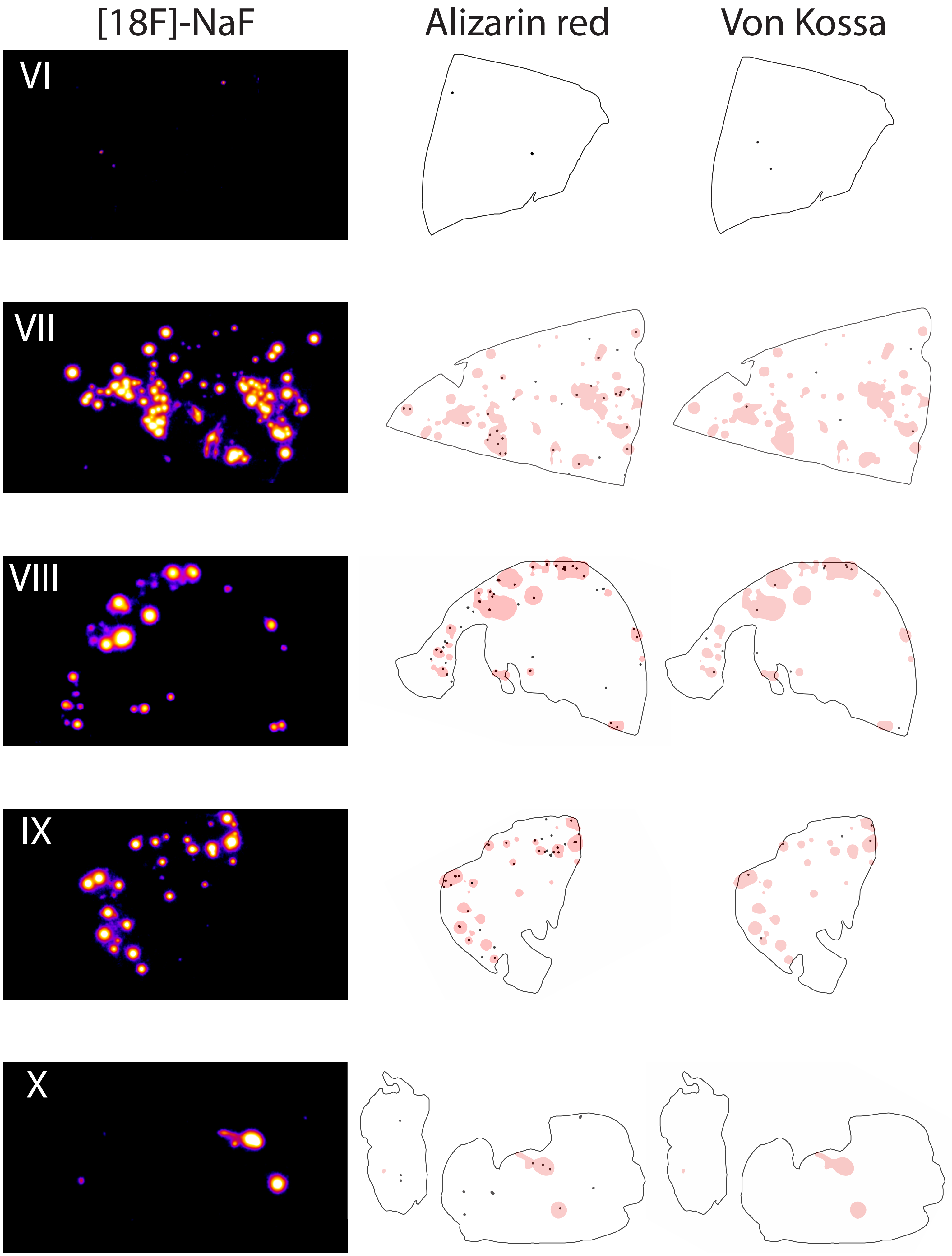

Supplementary S1

Kidney transplant recipient samples

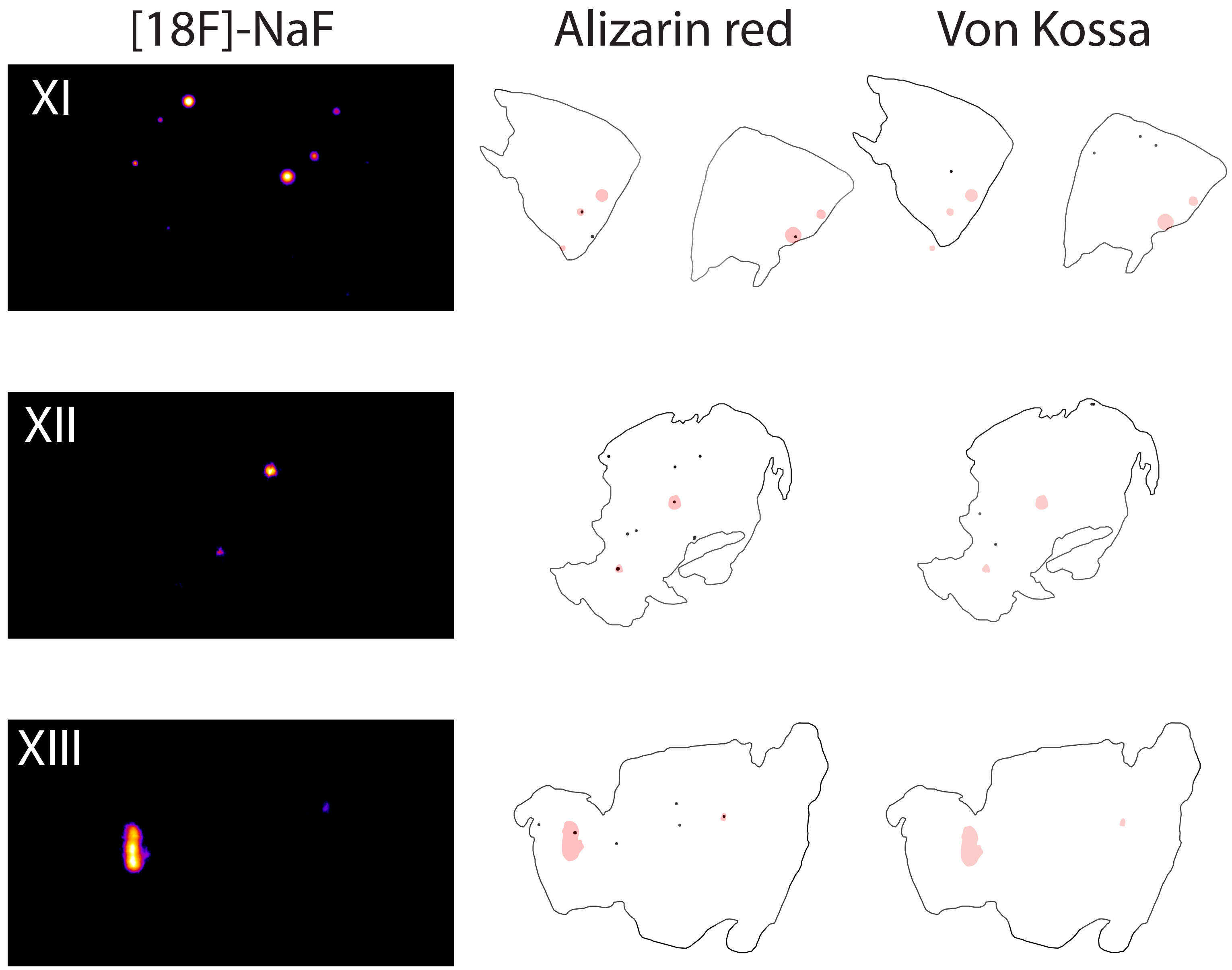

Supplementary S2
Deceased donor kidney samples

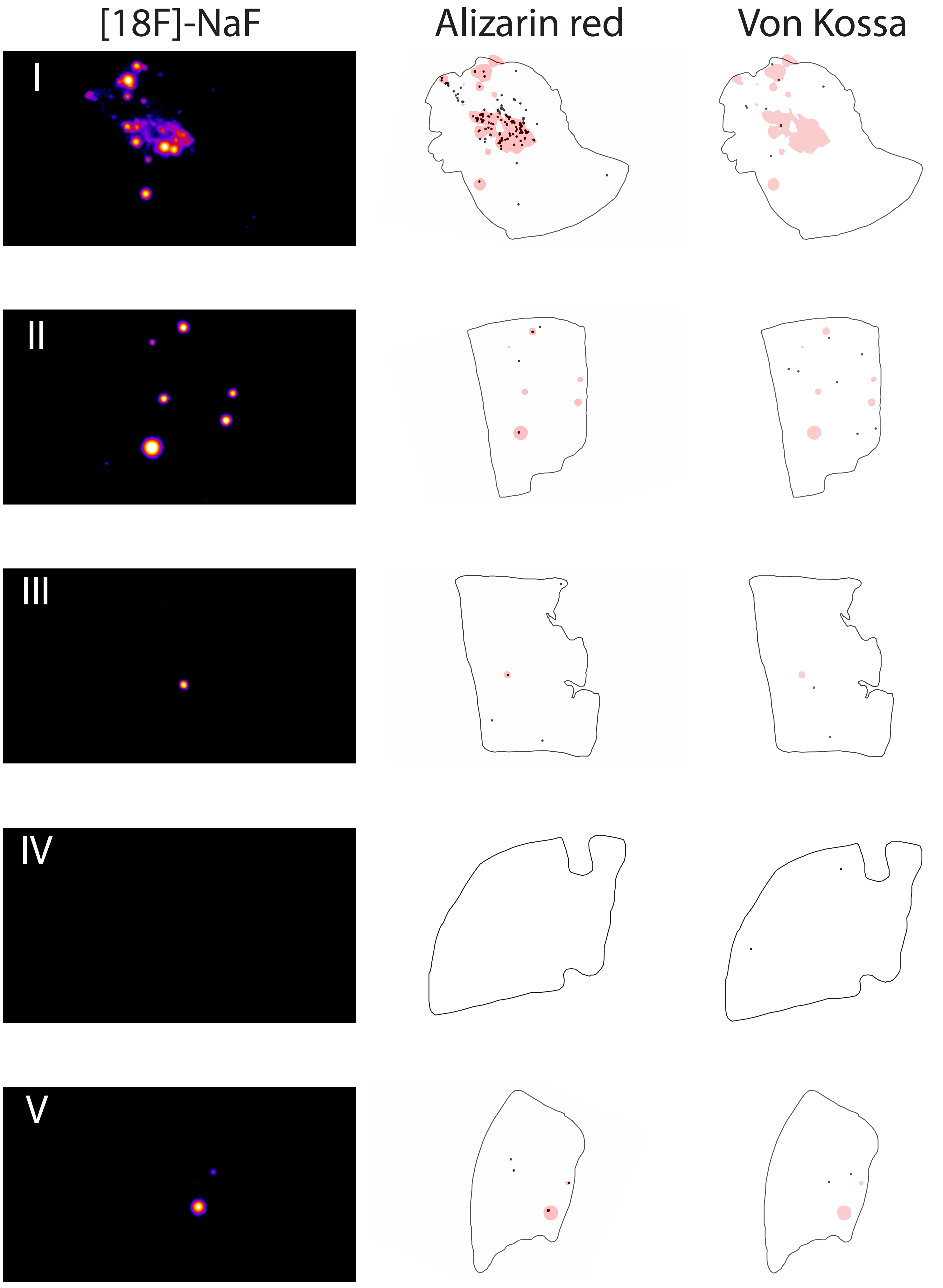

Supplementary S2
Deceased donor kidney samples

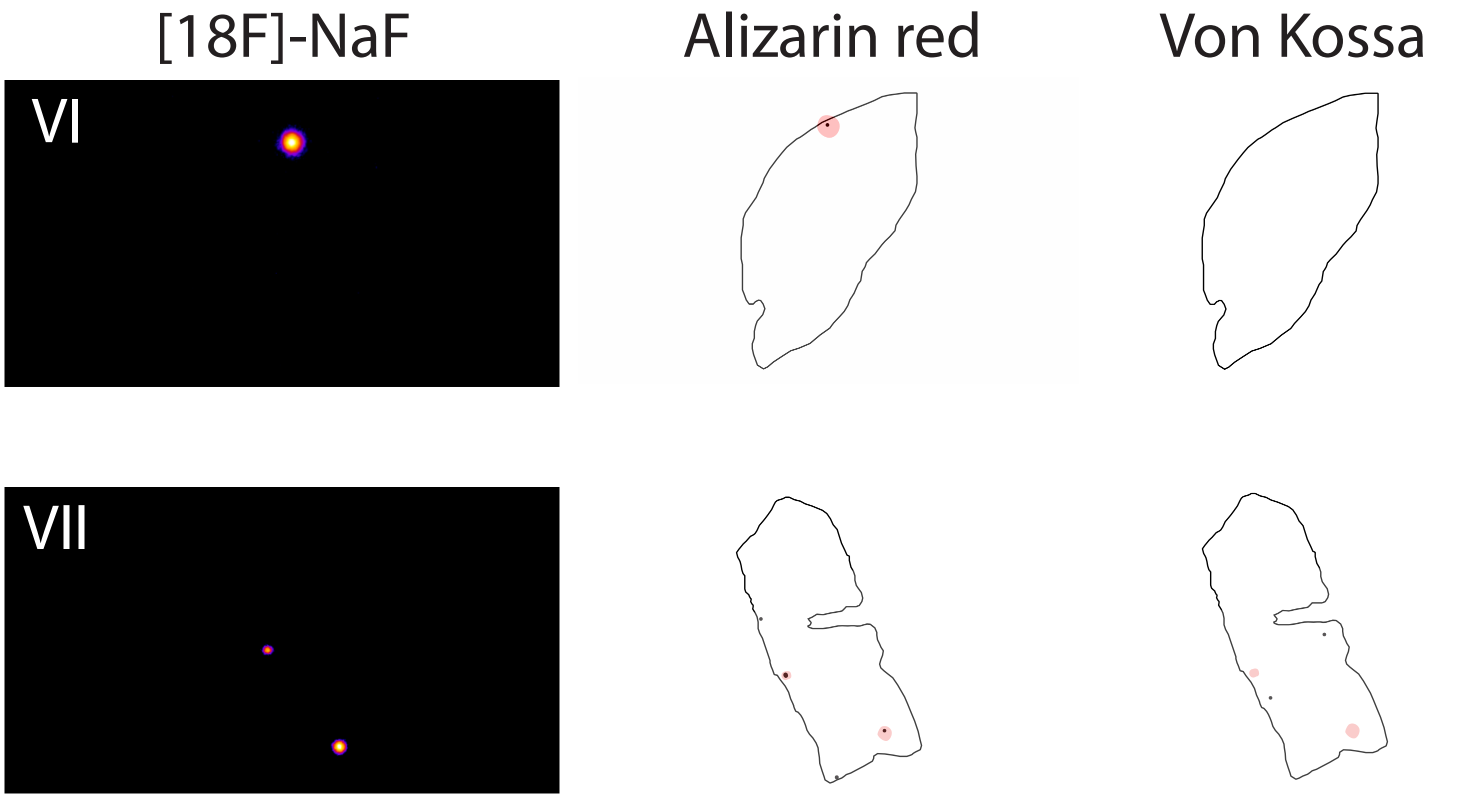

Supplementary S3

Differences between two groups, discarded deceased donor kidneys and explanted kidney allografts, for the number of [18F]-sodium fluoride ([18F]-NaF) areas (A), Alizarin red stained microcalcifications (B) and Von Kossa stained calcifications (C) (median, interquartile range).

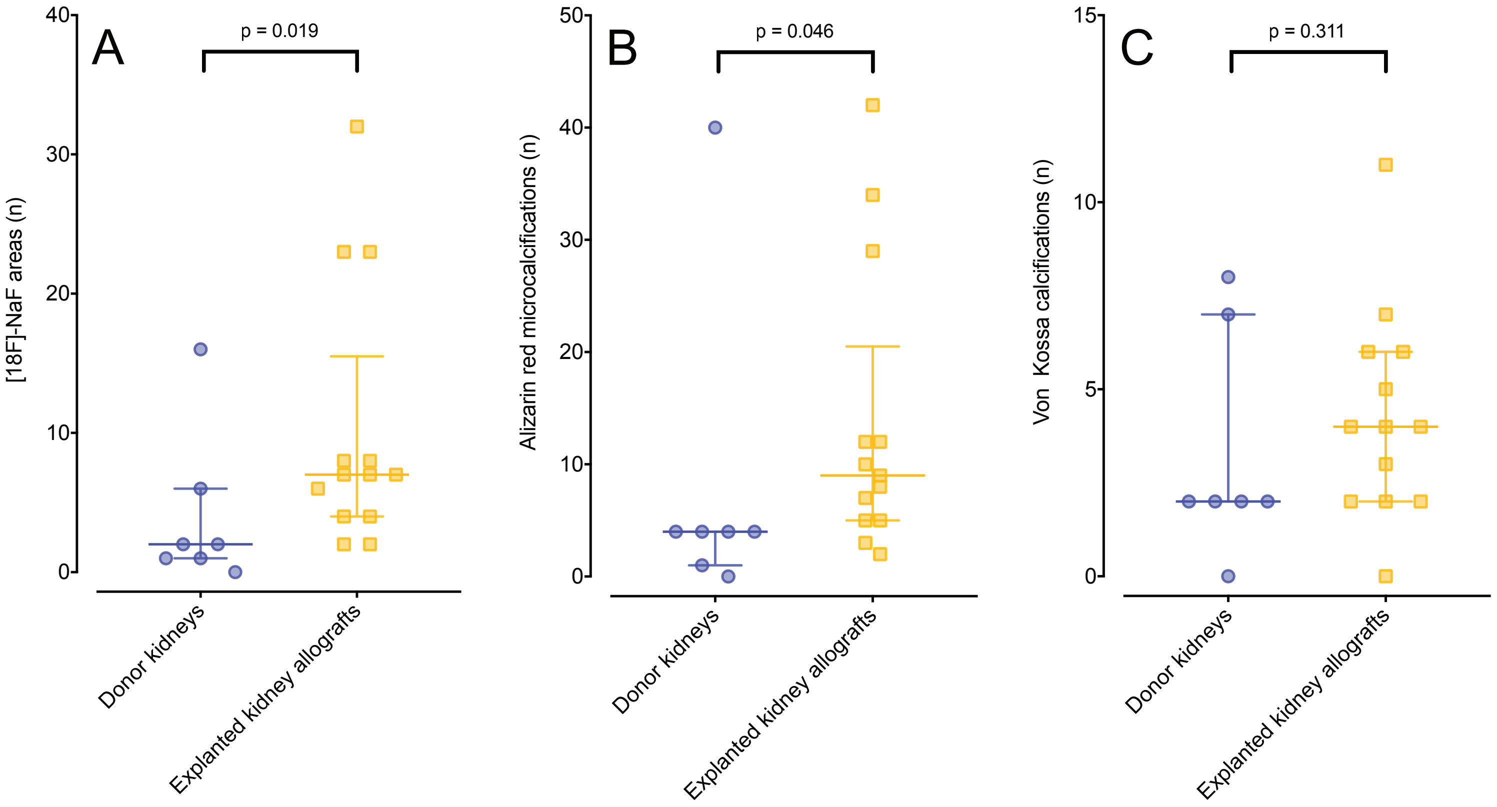

Supplement: Supplementary file 1 — Supplementary Figures. [file 41598_2021_81144_MOESM1_ESM.pdf]
